# The contribution of hormone sensitive lipase to adipose tissue lipolysis and its regulation by insulin in periparturient dairy cows

**DOI:** 10.1038/s41598-018-31582-4

**Published:** 2018-09-06

**Authors:** Jenne De Koster, Rahul K. Nelli, Clarissa Strieder-Barboza, Jonas de Souza, Adam L. Lock, G. Andres Contreras

**Affiliations:** 1 Department of Large Animal Clinical Sciences, Michigan State University, East Lansing, Michigan USA; 2 Department of Animal Science, Michigan State University, East Lansing, Michigan USA

## Abstract

Hormone sensitive lipase (HSL) activation is part of the metabolic adaptations to the negative energy balance common to the mammalian periparturient period. This study determined HSL contribution to adipose tissue (AT) lipolysis and how insulin regulates its activity in periparturient dairy cows. Subcutaneous AT (SCAT) samples were collected at 11 d prepartum (dry) and 11 (fresh) and 24 d (lactation) postpartum. Basal and stimulated lipolysis (ISO) responses were determined using explant cultures. HSL contribution to lipolysis was assessed using an HSL inhibitor (CAY). Basal lipolysis was higher in SCAT at dry compared with fresh. CAY inhibited basal lipolysis negligibly at dry, but at fresh and lactation it reduced basal lipolysis by 36.1 ± 4.51% and 43.1 ± 4.83%, respectively. Insulin inhibited lipolysis more pronouncedly in dry compared to fresh. Results demonstrate that HSL contribution to basal lipolysis is negligible prepartum. However, HSL is a major driver of SCAT lipolytic responses postpartum. Lower basal lipolysis postpartum suggests that reduced lipogenesis is an important contributor to fatty acid release from SCAT. Loss of adipocyte sensitivity to the antilipolytic action of insulin develops in the early lactation period and supports a state of insulin resistance in AT of cows during the first month postpartum.

## Introduction

Excessive release of fatty acids (FA) from adipose tissue (AT) is linked with metabolic diseases of gestation and early lactation in mammals^1,2^. Ketosis and fatty liver are prevalent in dairy cows with high concentrations of circulating FA throughout the periparturient period (3 wks before through 3 wks after calving)^3,4^. In humans and sheep, gestational diabetes mellitus or pregnancy toxemia mainly occur in the third trimester of pregnancy when circulating FA are increased considerably^5,6^. During the periparturient period, the net release of FA from AT into circulation is a result of reduced lipogenesis (including de novo lipogenesis and re-esterification) and enhanced lipolysis within adipocytes^7,8^. In humans and sheep, the majority of the FA reserves are mobilized before parturition. In contrast, seals, bears, and dairy cows mobilize these FA after parturition^7^. Excessive FA release during the periparturient period has been linked to alterations in lipolytic^9^ and lipogenic^10^ activity of adipocytes and changes in the response of adipocytes to regulatory hormones^11^. Consequently, excessive lipolysis is more common in animals with excessive fat accumulation, genetic predisposition, concurrent health disorders, inflammatory conditions, or malnutrition^4,12–15^. Endocrine factors regulate the FA release from AT. Catecholamines and growth hormone are the most important hormones increasing FA release while insulin is the most important hormone decreasing FA release^7,16^. These hormones act by stimulating or inhibiting the activity of hormone sensitive lipase (HSL) and adipose triglyceride lipase (ATGL) through phosphorylation by protein kinase A (PKA) or by changing the sensitivity of adipocytes to catecholamines^7,17^.

Currently, the exact contribution of lipolytic enzymes, lipogenic activity, and adipocyte insulin resistance to the net release of FA during the immediate periparturient period is not well established. A complete characterization of the lipolytic activity is important in species with a high incidence of gestational and periparturient diseases including humans, dairy cows, and other small ruminants. The present study aimed to determine how the lipolytic enzymes ATGL and HSL contribute to FA mobilization throughout the periparturient period using an adipose explant model in subcutaneous AT samples collected from Holstein Friesian dairy cows before and after calving. Also, the effect of insulin on lipolytic activity of AT was determined by supplementing insulin at physiological concentrations. Protein expression and activity of key enzymes (HSL and protein kinase B or AKT) in the regulation of AT metabolism was determined to identify potential altered pathways. Additionally, changes in the mRNA expression of important lipogenic, lipolytic, and glucose metabolism gene networks were determined.

## Materials and Methods

### Study design

All animal procedures were approved by the Michigan State University Animal Care and Use Committee. All experiments were performed in accordance with relevant guidelines and regulations. Twenty-two multiparous (3.14 ± 1.28 lactations, mean ± SD) Holstein dairy cows from the Michigan State University Dairy Teaching and Research Center (East Lansing, MI) were enrolled in this study. Cows were non-lactating and pregnant (>235 d of gestation). Body condition score (i.e. adiposity) was assessed at selection by three experienced technicians using a 5-point scale^18^. Cows were housed in tie-stall barns bedded with sawdust and fed a close-up diet from 3 wk before expected parturition date and a postpartum diet following parturition. All rations were formulated to meet or exceed the predicted requirements for protein, minerals, and vitamins according to NRC^19^. The ingredient and nutrient composition of the close-up diet and the postpartum diet are described in Supplementary Table A.

### Blood samples

Blood samples were collected weekly from 3 wk before expected parturition date until 3 wk after parturition. Blood was drawn before the morning feeding via coccygeal venipuncture using uncoated serum collection tubes, centrifuged for 20 min at 3,000 × *g* (15 °C) for serum fraction collection and then stored at −80 °C until further analysis. Serum concentrations of insulin, free fatty acids (FFA), and β-hydroxybutyrate (BHB) were determined using an Olympus AU640e chemistry analyzer (Olympus America, Center Valley, PA, USA) at the Diagnostic Center for Population and Animal Health of Michigan State University (Lansing, MI, USA).

### Adipose tissue samples

Subcutaneous AT (SCAT) samples were obtained from the right flank at 11 ± 1 d before expected parturition date (dry) and 11 ± 0.2 d (fresh) and 24 ± 0.4 d (lactation) after calving, using a surgical procedure described in detail by Mann, *et al*.^20^. Briefly, after local anesthesia (15 mL of 2% lidocaine hydrochloride 2%, VetTek, Middleburg, VA, USA) and aseptic preparation of the surgical field using iodine scrub, iodine prep, and alcohol, a vertical skin incision of 5 cm was made. Five grams of SCAT were collected. A part of the adipose sample was snap frozen in liquid nitrogen and stored at −80 °C until further analysis, a part was fixed in formaldehyde for 72 h, and a part was used for the *in vitro* lipolysis assay. The skin was closed using a continuous interlocking suture with Braunamid (USP1, Aesculap, Center Valley, PA, USA). Sutures were removed after 14 d.

### *In vitro* lipolysis assay

AT lipolysis was determined using a short-term *in vitro* explant culture as described by De Koster, *et al*.^9^. Krebs Ringer Bicarbonate HEPES buffer (KRBH, pH 7.4) containing 3% FA-free bovine serum albumin (BSA, Millipore-Sigma, Burlington, MA, USA) was prepared fresh on the day of the experiment. Immediately after sampling, AT was placed in 20 mL KRBH supplemented with 3% BSA and minced using scissors. Samples were stored and transported to the lab at 38 °C. Adipose explants (approximately 100 mg per culture dish) were incubated in 6 well plates containing 3 mL KRBH + 3% BSA on a shaker at 38 °C. After 20 min pre-incubation, reagents were added to the culture plates as described below. All reagents were prepared fresh on the day of the experiment. Basal lipolysis was determined without addition of any reagent. Stimulated lipolysis was determined by adding (-)-isoproterenol (ISO, I6504, Millipore-Sigma) at a concentration of 10^−6^ M. The inhibitory effect of insulin on the stimulated lipolytic activity was determined by adding insulin and (-)-isoproterenol (10^−6^ M) simultaneously to the culture dishes. Insulin was added at two different concentrations: 1 µg/L and 0.2 µg/L. The effect of CAY10499 (CAY, Cayman chemical, Ann Arbor, MI, USA, #10007875) on lipolytic activity was determined after adding CAY (dissolved in DMSO) to culture plates at a concentration of 2 µM. After 1 hour of pre-incubation with CAY, 2 different conditions were tested. To determine the effect of CAY on basal lipolytic activity, no reagents were added. To assess the effect of CAY on stimulated lipolytic activity, ISO was added at a concentration of 10^−6^ M. After 3 h of incubation, samples of medium were taken, snap-frozen in liquid N_2_ and stored at −80 °C until further analysis. The weight of the explants was determined precisely on an analytical scale (Mettler Toledo, Columbus, OH, USA, MS303TS/00) and the explants were snap frozen and stored at −80 °C for further analysis.

Lipolysis responses were assessed by glycerol concentrations released in the culture medium during the 3 h assay using free glycerol reagent (Millipore-Sigma, F6428). Intra-assay CV was 5.70%; inter-assay CV was 6.95%. Results of the lipolysis assays are expressed as glycerol release per million adipocytes per 3 hours. The number of adipocytes was determined as described by DiGirolamo and Fine^21^ using an Olympus BX-40 microscope (Olympus America). The diameter of the adipocytes (n = 100 per sample) was determined after digestion of 1 g of AT in 3 ml KRBH + 3% BSA containing 2 mg/mL collagenase type 2 (Worthington, Lakewood, NJ, USA) using ImageJ. Mean, and standard deviation of the diameter were used to calculate the volume of the adipocytes using the formula of Goldrick^22^. All conditions were performed in duplicate. Statistical analysis was performed using the average glycerol concentration of the duplicates. The intra-assay CV for the *in vitro* lipolysis assay was 10.4%. The effect of CAY on basal and ISO stimulated glycerol release was expressed as a percentage of the basal and ISO stimulated glycerol release, respectively. The effect of ISO on glycerol release was expressed as a percentage of the basal glycerol release, to correct for differences in basal glycerol release. The effect of insulin (1 µg/L and 0.2 µg/L) was expressed as a percentage of ISO stimulated glycerol release, to correct for differences in ISO stimulated glycerol release.

### RNA extraction from adipose samples

Snap-frozen SCAT samples weighing less than 200 mg were transferred into screwcap tubes containing 1 mL of TRizol reagent (Thermofisher Scientific, Waltham, MA, USA) and 2.3 mm zirconia/silica beads (Biospec, Bartlesville, OK, USA). The tubes containing tissues were placed in liquid nitrogen and then homogenized at 6,000 rpm for 3 times 30 sec using a bead mill homogenizer (Precellys, Bertin Instruments, Montigny-le-Bretonneu, France). Following homogenization, the TRizol supernatant was collected carefully, leaving the debris and beads at the bottom and avoiding the lipid layer on the top. After performing the chloroform phase separation, a commercially available kit (QIAGEN, Hilden, Germany) was used to extract total RNA according to the manufacturer’s protocol. Purity, concentration, and integrity of total RNA were evaluated using a NanoDrop 1000 spectrophotometer (Thermofisher Scientific) and an Agilent Bioanalyzer 2100 system (Agilent Technologies, Santa Clara, CA, USA). All samples had a 260:280 nm ratio between 1.96 and 2.01 and RNA integrity number >6. Reverse transcription was performed using the qScript cDNA SuperMix (Quantabio, Beverly, MA, USA).

### qRT-PCR analysis

Transcriptional studies were performed using the Wafergen SmartChip Real-time PCR system (Takara Bio, Mountain View, CA, USA) as described in^23^. Each run of SmartChip Real-time PCR system can perform 5,184 real-time PCR reactions with a volume of 100 nL each, and they were filled using the SmartChip Multisample Nanodispenser. Quality assurance and quality control checks were performed according to a standard protocol provided by Wafergen Biosystems. Each 100 nL PCR reaction contains 1X Applied Biosystems TaqMan Universal PCR Master mix (Thermofisher Scientific), 1X TaqMan gene expression assays (Supplementary Table B) and 1.5 ng/μL sample cDNA. The following cycling conditions were used on Wafergen SmartChip Real-time PCR system, initial enzyme activation at 95 °C for 10 min, 45 cycles of denaturation at 95 °C for 10 sec and annealing at 60 °C for 53 sec. Finally, qPCR results were analyzed using SmartChip qPCR software (v 2.8.6.1), an amplification efficiency beyond the range (1.5–2.2) and a threshold cycle (Ct) above 40 were discarded. All qPCR reactions were performed in duplicates, and no template controls (NTC) were included on each chip/plate for each TaqMan gene expression assay/ custom designed primers.

Gene expression data of 7 endogenous control genes (*ACTB*, *B2M*, *EIF3K*, *GAPDH*, *PPIA*, *RPLP0*, and *RPS9*) were analyzed using qBase+analysis software, which calculates the stability of endogenous control genes (M-value). Following qBase+analysis of gene expression data, endogenous control genes *RPS9, RPLP0* and *EIF3K* were ranked best. The Cq (quantification cycle) values of the target genes (*PNPLA2*, *ABDH5*, *LIPE*, *LPL*, *ACACA*, *FASN*, *ELOVL6*, *SCD1*, *AGPAT2*, *DGAT1*, *DGAT2*, *GLUT4* and, *PGK1*) were converted to normalized relative gene expression as described by Hellemans, *et al*.^24^. Information on the endogenous control genes and target genes can be found in Supplementary Table B.

### Western blot

A bead mill homogenizer was used to extract proteins from snap frozen SCAT samples weighing less than 100 mg using RIPA buffer (Teknova, Hollister, CA, USA) supplemented with cocktails of protease (Roche, San Francisco, CA, USA) and phosphatase (Thermofisher Scientific) inhibitors. A concentration of 0.2 mg/mL was found optimal to be used on all antibodies tested on 12–230 kDa Wes Separation Module capillary cartridges of Simple Protein Wes system (ProteinSimple, Santa Clara, CA, USA). Rabbit monoclonal antibody specific for total HSL (#4107, Cell Signaling, Danvers, MA, USA) and Akt (#9272 s, Cell Signaling) were used at a dilution of 1:50, while phosphorylated HSL (pHSL(Ser563), #4139t, Cell Signaling) was used at a dilution of 1:25 and phosphorylated Akt (pAkt (Ser473), #9271t, Cell Signaling) at a dilution of 1:10. A rabbit polyclonal antibody specific for vinculin (#4650, Cell signaling), a cytoskeletal protein, was used as a loading control (1:100 dilution). Anti-rabbit detection modules for Wes (ProteinSimple) kits include Luminol-S, Peroxide, antibody Diluent 2, Streptavidin-HRP and anti-rabbit secondary antibody. Sample proteins were allowed to separate by a capillary technology and were analyzed based on the chemiluminescence signal peaks generated, which were transformed into digital images depicting bands as observed in western blot analysis. Using Compass software (ProteinSimple), the peak areas of Akt, pAkt, HSL and pHSL proteins were estimated and normalized against vinculin. The peak areas are directly proportional to the amount of target protein. Protein abundance of total HSL and pHSL(Ser563) were determined in adipose explants from basal and CAY culture conditions. Protein abundance of total AKT and AKT phosphorylation were determined in adipose explants from basal and insulin stimulated (insulin 1 µg/L) culture conditions. Raw WES data were normalized to the area of vinculin. The normalized data are expressed as a ratio of phosphorylated protein over total protein (ratio pHSL(Ser563):HSL, ratio pAKT:AKT). The change in the ratio of phosphorylated protein by CAY and insulin (1 µg/L) was calculated as the difference in the ratio of phosphorylated protein between the basal sample and the CAY and insulin (1 µg/L) sample, respectively.

### Statistical analyses

Statistical analyses were performed using R^25^. Normality of the variables was checked using the Kolmogorov-Smirnov test (*P* < 0.05). Non-normally distributed variables (serum BHB and FFA concentrations, number of adipocytes per gram AT, gene expression data, ratio pHSL(Ser563):HSL, ratio pAKT:AKT) were ln-transformed. The effect of independent variables on the dependent variables was checked using a linear mixed effect model. Parity of the animals was included as a covariate in all models. Period or wk relative to the calving date was included as a repeated observation within the random factor cow. Two-way interactions were removed from the model if non-significant (*P* > 0.05). Pairwise comparisons were done using the Tukey’s post hoc test. Residuals of the models were checked and found to be normally distributed. Significance and tendency were declared at *P* < 0.05 and 0.05 < *P* < 0.10, respectively. Results are presented as LSMEANS ± SEM unless otherwise stated.

## Results

### Lipolysis during the periparturient period

**Figure 1:**
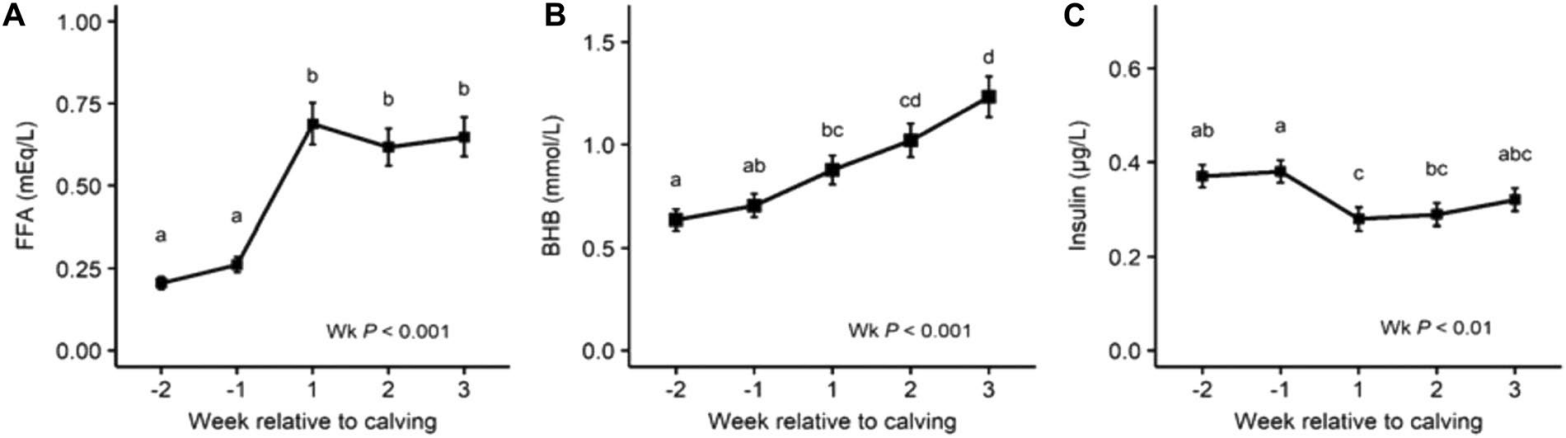
Serum free fatty acid (FFA), β-hydroxybutyrate (BHB) and insulin concentrations. Least squares means of FFA concentration (mEq/L) **(A)**, BHB concentration (mmol/L) **(B)** and insulin concentration (µg/L) **(C)** during the periparturient period. Error bars represent the SEM. Time-points with different letters differ significantly (abcd, *P* < 0.05). *P*-values for the effect of wk relative to calving on the different metabolites are derived from the linear mixed effect model.

**Figure 2:**
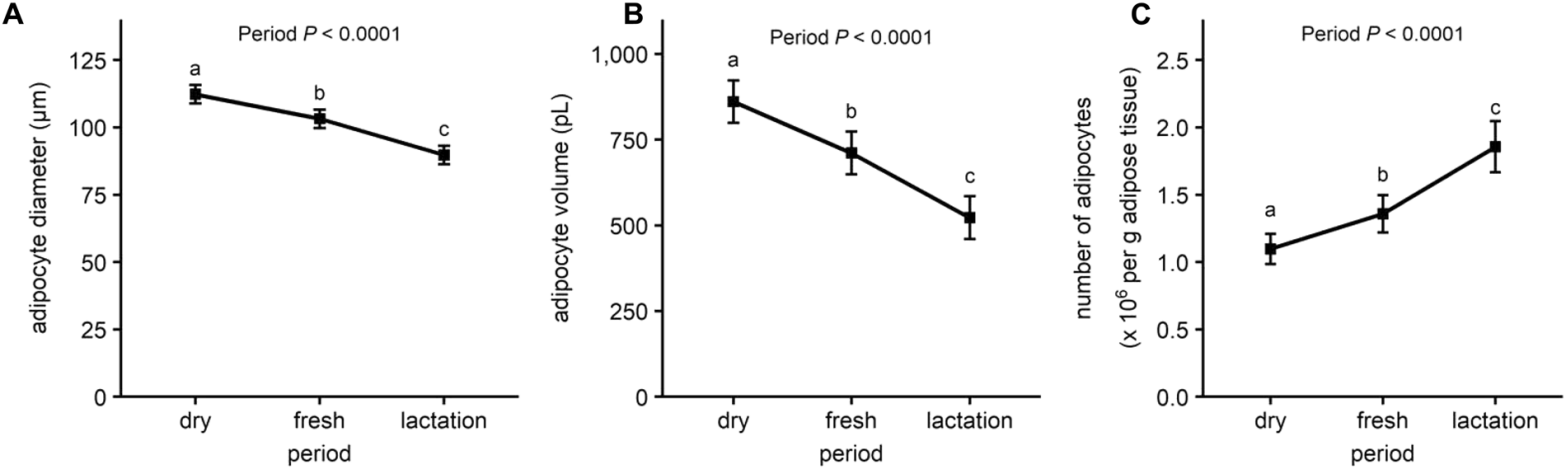
Adipocyte volume and diameter and number of adipocytes per gram adipose tissue. Least squares means of adipocyte diameter (µm) **(A)**, adipocyte volume (pL) **(B)** and number of adipocytes per gram AT (×10^6^ adipocytes per g AT) (C) in the SCAT samples at dry (−11 ± 1 d), fresh (+11 ± 0.2 d) and lactation (+24 ± 0.4 d relative to calving) from Holstein dairy cows. Error bars represent the SEM. Time-points with different letters differ significantly (abc, *P* < 0.05). *P*-values for the effect of period on the different variables are derived from the linear mixed effect model.

Reflecting the intense FA mobilization from AT during the periparturient period, serum FFA and BHB concentrations increased and body weight and body condition score were reduced in fresh and lactation compared with dry (Fig. 1 and Supplementary Table C). Insulin concentration decreased after parturition (fresh and lactation) compared to values observed at dry (Fig. 1). As expected, the diameter and volume of adipocytes decreased continuously throughout the experimental period. The volume of adipocytes was decreased by 40% at lactation compared with dry (Fig. 2). Due to the interrelationship between the size and the number of the adipocytes, the number of adipocytes per gram of AT increased at fresh and lactation compared with dry (Fig. 2).

### Basal lipolysis

**Figure 3:**
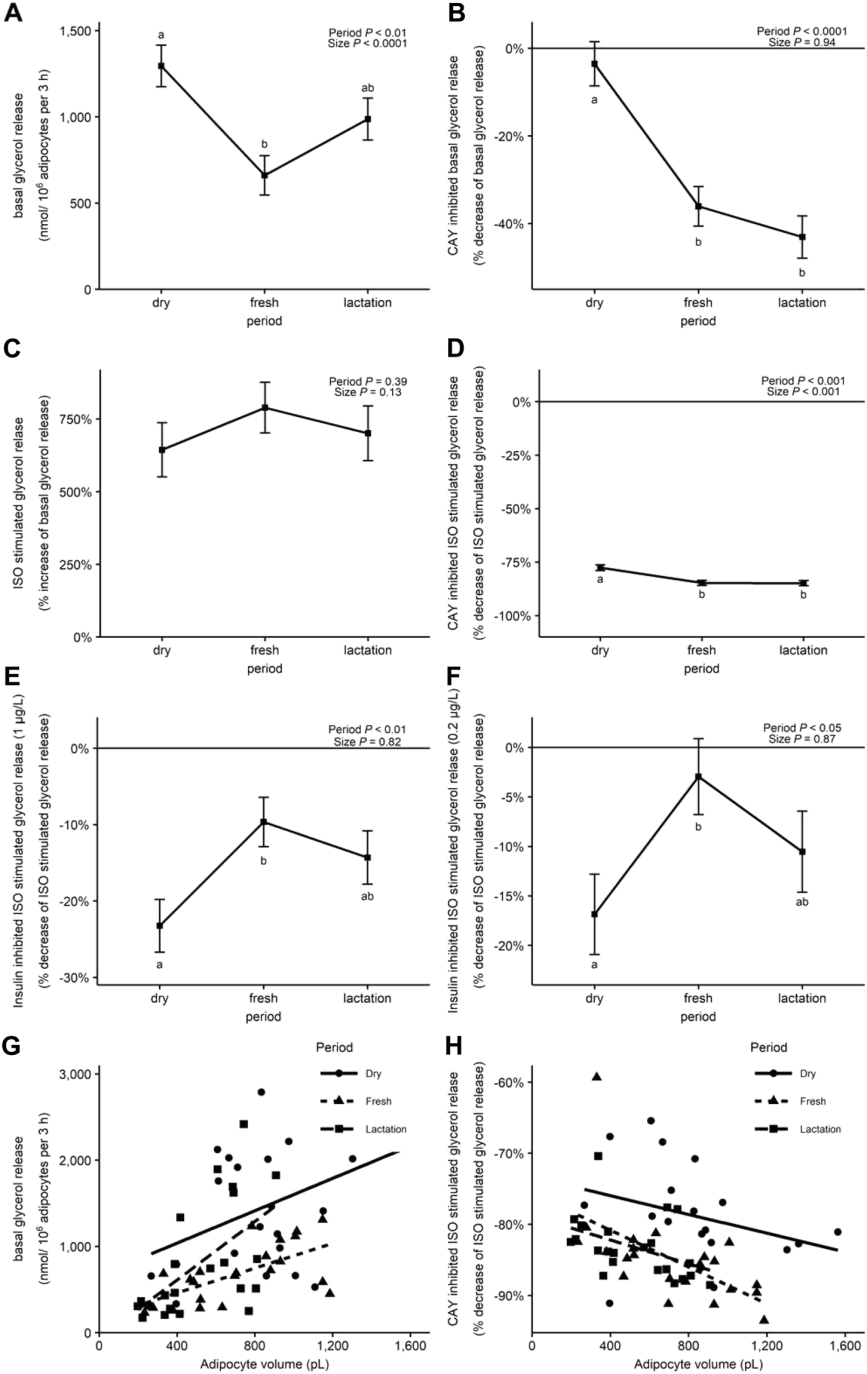
Glycerol release and the effect of adipocyte volume during inhibition of HSL activity. Least squares means of basal glycerol release (nmol/10^6^ adipocytes per 3 h) **(A)**, CAY inhibited basal glycerol release (% decrease of basal glycerol release) **(B)**, isoproterenol (ISO) stimulated glycerol release (% increase of basal glycerol release) **(C)**, CAY inhibited ISO stimulated glycerol release (% decrease of ISO stimulated glycerol release) **(D)** and insulin (0.2 and 1 µg/L) inhibited ISO stimulated glycerol release (% decrease of ISO stimulated glycerol release) **(E**,**F)**. Effect of adipocyte volume on basal glycerol release **(G)** and CAY inhibited ISO stimulated glycerol release **(H)** in SCAT explants from Holstein dairy cows at dry (−11 ± 1 d), fresh (+11 ± 0.2 d) and lactation (+24 ± 0.4 d relative to calving). Error bars represent the SEM. Time-points with different letters differ significantly (ab, *P* < 0.05). *P*-values for the effect of period and adipocyte size (volume in pL) on the different variables are derived from the linear mixed effect model.

Basal lipolytic activity was higher in adipose explants from dry compared with fresh (Fig. 3A). In adipose samples taken at lactation, basal lipolytic activity was numerically increased compared with those taken at fresh (Fig. 3A, *P* = 0.13). Independent of the period, explants with larger adipocytes demonstrated higher basal lipolytic activity (Fig. 3G). Inhibition of basal lipolytic activity by CAY was minimal at dry (Fig. 3B). In fresh and lactation, CAY inhibited basal lipolysis by 36.05 ± 4.51% and 43.05 ± 4.83%, respectively (Fig. 3B).

### Stimulated lipolysis is affected by adipocyte size and time relative to parturition

The beta-adrenergic agonist ISO stimulated the lipolytic activity of adipose explants in all periods in a similar way (Fig. 3C). Compared with basal lipolysis, ISO increased lipolysis 5- to 10-fold (Fig. 3A,C). Inhibition of HSL activity by CAY during ISO stimulation was more pronounced in the fresh and lactation compared with dry (Fig. 3D). The inhibition of stimulated lipolytic activity by CAY was more pronounced in larger adipocytes (Fig. 3H, *P* < 0.001).

### Insulin inhibition of lipolytic activity is reduced after calving

Insulin inhibited ISO stimulated lipolytic activity in SCAT explants, and the inhibition was more pronounced for the 1 µg/L compared with 0.2 µg/L insulin dose (Fig. 3E,F). Inhibition of stimulated lipolysis by insulin was more marked in dry compared with fresh for both insulin doses (Fig. 3E,F). Adipocyte size did not influence the inhibitory effect of insulin.

### Lipolytic and lipogenic gene networks after parturition

Expression of the lipolytic gene network was paradoxically reduced during the peak of FA mobilization postpartum. AT expression of *ABDH5*, *LIPE*, *LPL*, and *PNPLA2* (encoding ATGL) decreased in fresh and lactation compared with samples taken at dry (Table 1). The expression of *LPL* was decreased pronouncedly in fresh and lactation compared to dry (Table 1).

**Table 1:** mRNA expression^a^ of *PNPLA2, ABDH5, LIPE, LPL, ACACA, FASN, ELOVL6, SCD1, AGPAT2*, *DGAT1, DGAT2, GLUT4* and, *PGK1*.

	Period^a^			*P* value^b^		
	Dry	Fresh	Lactation	Period	Size	Period*Size
**Lipolytic gene network**						
*PNPLA2*	2.02 ± 0.32^a^	0.99 ± 0.15^b^	0.47 ± 0.08^c^	<0.0001	0.49	ns
*ABDH5*	1.55 ± 0.30^a^	0.67 ± 0.13^b^	0.81 ± 0.16^ab^	<0.01	0.82	ns
*LIPE*	1.31 ± 0.25^a^	1.26 ± 0.23^a^	0.58 ± 0.11^b^	<0.01	0.97	ns
*LPL*	3.63 ± 0.74^a^	0.63 ± 0.12^b^	0.34 ± 0.07^c^	<0.0001	0.31	ns
**Lipogenic gene network**						
*ACACA*	3.95 ± 0.61^a^	0.44 ± 0.07^b^	0.46 ± 0.07^b^	<0.0001	0.43	ns
*FASN*	8.55 ± 2.00^a^	0.26 ± 0.06^b^	0.37 ± 0.09^b^	<0.0001	0.21	ns
*ELOVL6*	3.84 ± 0.88^a^	0.54 ± 0.12^b^	0.35 ± 0.09^b^	<0.0001	0.55	ns
*SCD1*	17.33 ± 5.18^a^	0.25 ± 0.07^b^	0.19 ± 0.06^b^	<0.0001	0.14	ns
*AGPAT2*	4.78 ± 1.09^a^	0.70 ± 0.15^b^	0.30 ± 0.07^c^	<0.0001	0.91	ns
*DGAT1*	0.88 ± 0.10	0.95 ± 0.10	1.02 ± 0.12	0.63	0.29	ns
*DGAT2*	6.71 ± 1.56^a^	0.43 ± 0.09^b^	0.40 ± 0.11^b^	<0.0001	0.05	0.08
**Glucose metabolism gene network**						
*SLC2A4*	1.62 ± 0.26^a^	0.84 ± 0.13^b^	0.59 ± 0.10^b^	<0.001	0.05	ns
*PGK1*	1.53 ± 0.12^a^	0.86 ± 0.07^b^	0.75 ± 0.06^b^	<0.0001	0.71	ns

Least squares means ( ± SEM) of the mRNA expression of selected genes of the lipolytic, lipogenic, and glucose metabolism gene networks in SCAT samples taken during the dry (−11 ± 1 d), fresh (+11 ± 0.2 d) and lactation (+24 ± 0.4 d relative to calving) period from Holstein dairy cows.

^abc^Time-points with different letters differ significantly (*P* < 0.05).

^a^mRNA expression of the genes is presented as relative mRNA abundance after normalization with the reference genes (*EIF3K*, *RPLPO*, *RPS9*).

^b^*P*-values for the effect of period and adipocyte size (volume in pL) on the mRNA expression of the different genes are derived from the linear mixed effect model.

**Figure 4:**
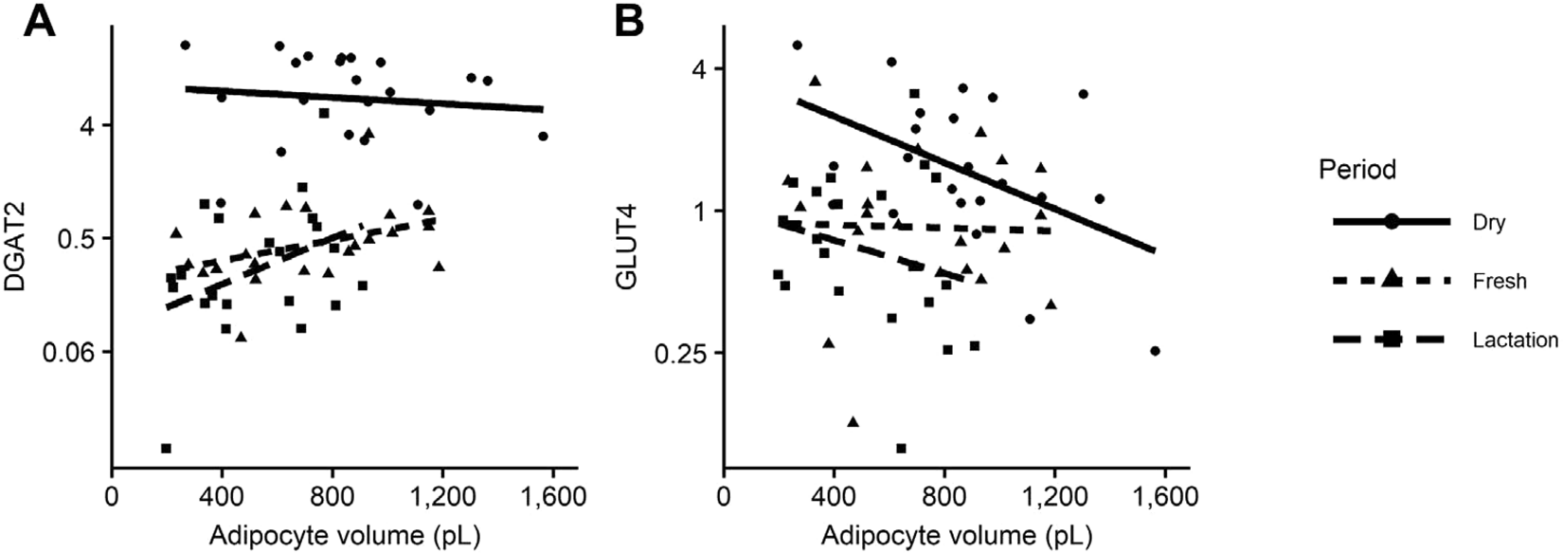
Effect of adipocyte volume on the mRNA expression of *DGAT2* **(A)** and *GLUT4* **(B)** in SCAT samples. mRNA expression of the genes is presented as relative mRNA abundance after normalization with the reference genes (*EIF3K*, *RPLPO*, *RPS9*).

Similarly, the lipogenic gene network was downregulated after parturition (fresh and lactation). The expression of genes involved in the FA synthesis process of the de-novo lipogenic pathway such as ACACA and FASN decreased dramatically in AT samples taken after calving (fresh and lactation) compared with dry (Table 1). Genes of the glycerol-3-phosphate pathway that are part of the triacylglycerol synthesis process including AGPAT2, DGAT2 and those encoding enzymes related to the elongation and desaturation of FA such as ELOVL6 and SCD1 decreased profoundly after calving (fresh and lactation) (Table 1). Remarkably, the expression of DGAT1 was not affected by period (Table 1). Higher expression of DGAT2 was associated with larger adipocytes in adipose samples at fresh and lactation samples but not in the dry samples (Fig. 4). The expression of two genes encoding important proteins from the gene network involving the metabolism of glucose, SLC2A4 (formerly known as GLUT4 or glucose transporter 4) and PGK1, were decreased in fresh and lactation compared with the dry sample (Table 1).

### HSL and AKT activity in periparturient cows

**Figure 5:**
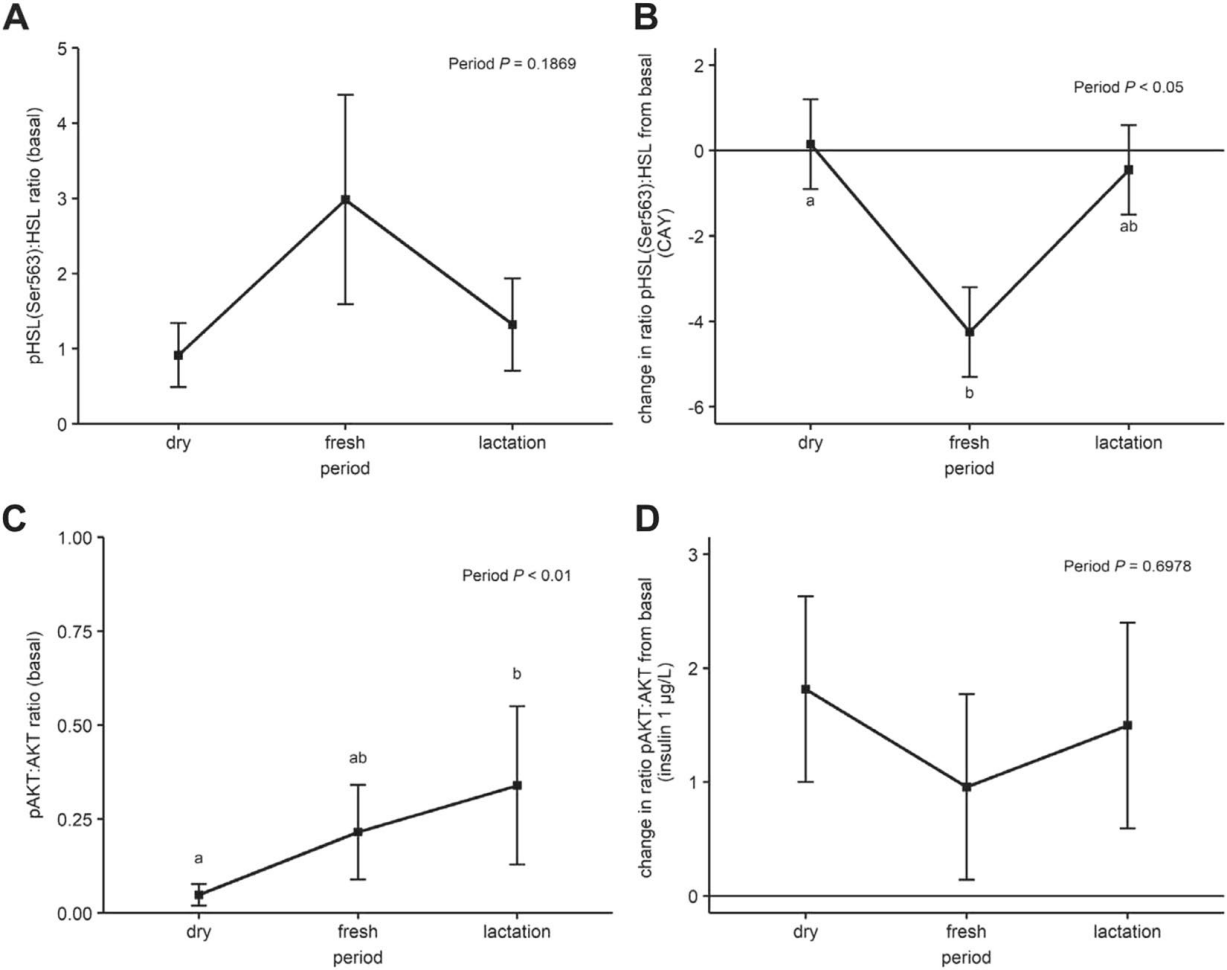
HSL and PKA activity in periparturient cows. Least squares means of the ratio of pHSL(Ser563):HSL **(A)** and pAKT:AKT **(C)** in explants cultured in basal incubation condition from SCAT samples taken during the dry (−11 ± 1 d), fresh (+11 ± 0.2 d) and lactation (+24 ± 0.4 d relative to calving) period from Holstein dairy cows. Change in ratio of pHSL(Ser563):HSL in explants incubated with CAY compared with basal incubation conditions **(B)** and change in ratio of pAKT:AKT in explants incubated with insulin (1 µg/L) compared with basal incubation conditions **(D)**. Error bars represent the SEM. Time-points with different letters differ significantly (ab, *P* < 0.05). *P*-values for the effect of period on the ratio of protein phosphorylation and the change in ratio of protein phosphorylation are derived from the linear mixed effect model.

HSL content in AT did not change over time in the basal samples: the ratio of HSL:vinculin was 0.50 ± 0.23, 0.24 ± 0.11, 0.50 ± 0.24 in the dry, fresh and, lactation periods, respectively (*P* value for the period effect = 0.39). The ratio of pHSL(Ser563):HSL reflected the HSL activation of the explants and was numerically increased in the fresh period compared with the dry and lactation period (Fig. 5A). CAY decreased the ratio of pHSL(Ser563):HSL especially in the fresh period, when the ratio of phosphorylation of HSL was maximal (Fig. 5B).

The total content of AKT in AT decreased over time: the ratio of AKT:vinculin was 1.49 ± 0.57, 0.36 ± 0.14, 0.55 ± 0.21 in the dry, fresh and, lactation period, respectively (*P* value for the period effect < 0.01). The ratio of pAKT:AKT reflects the activation of the insulin signaling cascade of the explants. There was an increase in the ratio of pAKT:AKT in the basal samples taken during the fresh and lactation period (Fig. 5C). Addition of insulin (1 µg/L) to the explants increased the ratio of pAKT:AKT. The effect of insulin on the activation of AKT was more pronounced in the dry and lactation period compared with the fresh period (Fig. 5D).

## Discussion

Mobilization of FA reserves from AT supports energy deficits generated by rapid fetal growth and lactation during the periparturient period. In the present study, net FA release from adipocytes into circulation was reflected by an increase in plasma BHB and FFA concentrations, a reduction in the diameter and volume of adipocytes, and an increase in adipocyte number per g of tissue^26^. The 40% decrease in volume of the adipocytes between the dry and lactation periods observed in our experiment is in agreement with previous studies^26,27^.

Our results demonstrate that the level of basal lipolytic activity in SCAT is dynamic throughout the periparturient period and decreases in the fresh compared with the dry period. Similarly, Kokkonen, *et al*.^28^ and Kenez, *et al*.^29^ observed a decrease in basal lipolysis in the immediate postpartum period. Other studies reported that basal lipolysis did not increase substantially after calving^30,31^. Although our observation of lower basal lipolysis postpartum is in contrast with the general perception that lipolysis increases after parturition, during this time higher concentrations of BHB exert an inhibitory effect on lipolysis via the G protein-coupled receptor 109 A (GRP109A)^32^. As lactation progresses, this inhibitory effect declines due to reductions in blood BHB concentrations. Additionally, results from the present study indicate that the postpartum peak in circulating FFA may be related to the drastic reduction in the lipogenesis process, that includes re-esterification of FFA, rather than an increase in lipolysis^33^. Although AT explant lipogenic activity was not measured in the present study, the mRNA expression of the lipogenic network decreased after parturition supporting our hypothesis. It is important to note that the present study did not evaluate basal lipolysis in visceral AT. Thus it is possible that, given the substantial differences in lipolytic activity between adipocytes from different depots^9,34^, circulating FFA could be derived from internal adipose depots rather than SCAT.

CAY is a known inhibitor of HSL activity in murine adipocytes^35^. In humans, CAY also appears to have an additional inhibitory effect on ATGL and monoacylglycerol lipase^36^. As far as the authors are aware, this is the first study using pharmacological inhibition of HSL in explants from dairy cattle. The observed effect of CAY on ISO stimulated lipolysis suggests an important inhibitory effect of CAY on HSL activity in bovine adipocytes that is supported by decreased phosphorylation of HSL on serine 563 in the explants treated with CAY. The absence of any effect of CAY on basal lipolysis in the dry period may suggest that CAY does not affect ATGL activity in bovine adipocytes or that the activity of ATGL and HSL is so low that it cannot be further reduced. Based on our results we cannot exclude any effect of CAY on MGL. However, MGL activity accounts for only 5% of lipolysis in murine AT^37^.

Based on the effects of CAY on basal and stimulated lipolysis, and pHSL(Ser563):HSL, we hypothesize that HSL contribution to basal lipolysis is dynamic during the transition period. In the dry period, we observed that HSL activity is of minor importance and most of the glycerol release was due to ATGL activity. In the fresh and lactation periods, nearly half of the basal lipolysis was attributable to HSL activity. These observations are in agreement with the increased rate of HSL phosphorylation in the postpartum period despite a decrease in total HSL protein content^20,29^. Interestingly, basal lipolysis (combined ATGL and HSL activity) was positively related with adipocyte size while the effect of CAY (only HSL activity) on basal lipolysis was not. It is known that basal lipolysis is related to adipocyte size^9,38^ and regulated by ATGL activity^39,40^. Overfed cows, which are expected to have larger adipocytes, had higher expression of ATGL but not of HSL^11,41^. In most studies, ATGL protein content or gene expression remain unchanged or downregulated in early lactation and are independent of energy balance status^40,42,43^. In our study, this was reflected by the decrease in ATGL dependent basal lipolytic activity (i.e. residual basal lipolytic activity in the presence of CAY). In agreement with Koltes and Spurlock^40^, this finding reflects a shift from ATGL dependent basal lipolysis during positive energy balance (dry period) to HSL dependent basal lipolysis during negative energy balance (fresh and lactation period). The increase in HSL activity postpartum is caused by different mechanisms including decreased insulin concentrations, potential decreased insulin signaling in adipocytes, increased growth hormone concentrations, and increased catecholamine sensitivity of the adipocytes^7,44,45^. Based on these observations, we suggest that ATGL determines a certain level of basal lipolytic activity that is directly related to adipocyte size. HSL modulates the level of lipolysis based on the individual requirements of each animal^44^. Future studies inhibiting ATGL activity may elucidate the role of this lipase in modulating basal lipolysis in dairy cows.

In adipocytes, stimulated lipolysis is triggered by catecholamines signaling through β adrenergic receptors that in turn activate HSL and ATGL^37,40,46^. HSL is more responsive to catecholamines compared with ATGL^44^. In our study, ISO, a non-selective β agonist, increased lipolysis independently of the period when biopsies were collected. Around 80% of the ISO stimulated lipolytic activity was due to HSL as indicated by the inhibitory effect of CAY on ISO stimulated glycerol release. This finding is in contrast with the observation that lipolytic responses of adipocytes to catecholamines is increased in the postpartum period^47^. Based on our calculations of the contribution of HSL to both basal and demand lipolysis, we propose that higher basal HSL activity (i.e., the inhibitory effect of CAY on basal glycerol release) in the fresh and lactation period explains in part the higher inhibitory effect of CAY on ISO stimulated lipolysis postpartum.

Insulin is the most potent physiological inhibitor of lipolysis in AT^48^. The effect of insulin on stimulated lipolysis was determined at insulin doses reflecting physiological insulin concentrations of dry (1 µg/L)^49^ and fresh (0.2 µg/L)^11,50^ periods of dairy cows. Since insulin exerts an inhibitory effect on stimulated lipolysis, by decreasing HSL phosphorylation, it was necessary to determine the effect of insulin after ISO treatment^51^. Inhibition of ISO stumulated lipolysis by both doses of insulin was less pronounced in the fresh period compared with the dry period. Also, protein phosphorylation of AKT, measured as pAKT:AKT, was reduced in the fresh period compared with the dry and lactation period. These observations indicate that during the fresh period adipocytes exhibit limited sensitivity to the anti-lipolytic actions of insulin. This insulin resistant state appears to be limited in time since the anti-lipolytic effect of insulin increased during the lactation period. Similar observations were reported by Ji, *et al*.^11^ in AT collected from cows at 1 and 3 weeks after parturition using insulin at supra-physiological concentrations (1 µM). Insulin signaling in adipose samples taken during intravenous glucose tolerance tests was lower in the postpartum period compared with the prepartum period^20,52^. However, it is important to clarify that insulin concentrations during intravenous glucose tolerance tests are usually supra-physiological and typically a reduction in insulin secretion is observed in the postpartum period.

This is the first study to demonstrate an insulin resistant state in adipocytes from cows during the first week of lactation (fresh) using physiologically relevant insulin concentrations. In the immediate postpartum period, minimal lipolysis inhibition and reduced lipogenesis stimulation driven by low insulin concentrations and reduced sensitivity to the anti-lipolytic effects of insulin increase the net release of FA into circulation. Insulin resistance during the first 2 weeks after parturition might support a sufficient FA release from AT while adipocytes adapt their metabolism to the intense energy requirements of lactation. However, AT insulin resistance in the immediate postpartum period may have a detrimental impact on health and productivity of dairy cows because it promotes excessive lipolysis^52^. Further research is needed to determine the physiological and pathological consequences of periparturient adipocyte insulin resistance.

Insulin inhibits lipolytic activity by decreasing the phosphorylation and thus activity of HSL. After insulin binds to its receptor, intracellular signaling is activated via phosphorylation of insulin receptor substrate 1 (IRS1), PI 3-kinase (PI3K), AKT, and the activity of phosphodiesterase 3b (PDE3b)^53,54^. *In vitro* studies using adipocyte cell lines demonstrated that, depending on the metabolic environment, the anti-lipolytic effect of insulin occurs via an AKT-dependent or an AKT-independent pathway. At high concentrations of ISO, maximal HSL activity, insulin inhibits lipolysis by an AKT-dependent pathway. At lower ISO concentrations, lipolysis is inhibited in an AKT-independent pathway^55^. In the present study, ISO dose (1 µM) was chosen to maximize adipocyte lipolytic activity before and after parturition^56^. We conclude that since ISO stimulated lipolysis was not affected by time relative to parturition, the anti-lipolytic effect of insulin is directly related to a state of insulin resistance in the adipocyte. Given that insulin only inhibits lipolysis through the reduction of HSL phosphorylation, it would not be possible to determine the inhibitory effect of insulin on basal lipolysis at dry when HSL activity was minimal (i.e., low ratio of pHSL(Ser563):HSL). Hence it is necessary to standardize the lipolytic stimulus before insulin responses can be assessed *in vitro* explants.

In the present study, expression of lipolytic genes (*PNPLA2*, *ABDH5*, *LIPE*, and *LPL*) decreased after calving. In line with these results, other groups reported lower protein expression of ATGL, HSL and ABHD5 (encoded by *PNPLA2*, *LIPE*, and *ABDH5* respectively) during early lactation compared to the dry period^30,40,57^. Accordingly, in the present study, total HSL protein expression was lower in the fresh period compared with the dry and lactation periods. In a study of Koltes and Spurlock^40^, protein expression of ATGL decreased in early lactation. And other studies have demonstrated that AT lipolysis is mainly regulated by posttranslational control mechanisms, i.e., phosphorylation of key enzymes, and related to milk production^30,31,58^. The decreased protein expression of HSL is accompanied by an increase in the ratio of phosphorylated HSL in the fresh period, indicating an increased lipolytic activity of HSL^57^.

Basal glycerol release was influenced by adipocyte size. Larger adipocytes had higher basal lipolytic activity compared with smaller adipocytes independent of the period when the samples were collected. Contrary, mRNA expression of lipogenic genes (except *DGAT2*) was not affected by adipocyte size. The imbalance between the lipolysis and lipogenesis in larger adipocytes favoring lipolytic activity in the basal state may provide an explanation why over-conditioned cows, which have larger adipocytes^9^, are at increased risk of having higher concentrations of circulating FFA and BHB during the periparturient period^59,60^. Remarkably, the anti-lipolytic effect of insulin was not influenced by adipocyte size as previously shown in over-conditioned cows^9^ which indicates that stimulated lipolysis in adipocytes from over-conditioned cows is not insulin resistant. More research is needed to substantiate the effect of lipogenic enzymes in the postpartum period and to determine the potential effect of modulating lipogenic activity on FA mobilization from AT.

Despite being lipolytic, LPL is responsible for the provision of FA to the adipocytes by breaking down circulating triacylglycerol in the capillaries of AT^37^. Decreased expression of LPL is thus an indication of decreased availability of FA for lipogenesis in AT^11^. The activity of lipogenic enzymes is controlled by transcriptional mechanisms and affected by the availability of energy^11,58^. Negative energy balance at the beginning of lactation leads to a pronounced decrease in the expression and activity of genes encoding proteins of the de-novo lipogenic, and glycerol-3-phosphate pathways^7,11,29^. Reduced lipogenic activity in AT may contribute to the increase in circulating FFA levels in the immediate postpartum period^33^. More research is warranted to determine the impact of modulating lipogenic enzyme activity on FA release from AT in the periparturient period of dairy cows.

The decreased expression of genes for glucose metabolism, *GLUT4*, and *PGK1*, in the postpartum period, is in agreement with the homeorhetic mechanisms that preserve glucose for milk production. In the postpartum period, lower *PGK1* will lead to lower glycolysis within adipocytes. And at the same time, downregulation of *GLUT4* expression leads to lower insulin-dependent glucose uptake in the adipocytes^11,61^. *GLUT4* expression was specially reduced in large adipocytes during the dry period. This may indicate that the glucose metabolism of large adipocytes is less sensitive to insulin compared with small adipocytes prior to calving. The abundance of GLUT4 protein was lower in SCAT samples of over-conditioned cows in the prepartum period but not in the postpartum period^61^. Similarly, insulin sensitivity of glucose metabolism was negatively associated with fat accumulation in dairy cows at the end of the dry period^62^. These results provide further evidence of an insulin resistant state of glucose metabolism of adipocytes by excessive accumulation of triacylglycerol through a decreased expression of *GLUT4*.

## Conclusion

AT lipolytic responses during the periparturient period are characterized by a shift from ATGL dependent basal lipolysis during positive energy balance (dry period) to HSL dependent basal lipolysis during negative energy balance (fresh and lactation period). The decrease in basal lipolytic activity and a broadly decreased transcriptional regulation of the lipogenic gene network suggests that decreased lipogenesis is an important contributor to FA release from SCAT postpartum. In addition, insulin resistance of lipolytic activity develops during the early lactation period and is characterized by a decreased activation of AKT.

## Electronic supplementary material

Supplementary Tables
