# Supplementary material for: The contribution of hormone sensitive lipase to adipose tissue lipolysis and its regulation by insulin in periparturient dairy cows
Source: Sci Rep. 2018 Sep 6;8:13378. doi: 10.1038/s41598-018-31582-4 (PMC6127149; doi:10.1038/s41598-018-31582-4)
Supplement: Supplementary file 1 — Supplementary Tables [file 41598_2018_31582_MOESM1_ESM.docx]

**The contribution of hormone sensitive lipase to adipose tissue lipolysis and its regulation by insulin in periparturient dairy cows**

Jenne De Koster^1^, Rahul K Nelli^1^, Clarissa Strieder-Barboza^1^, Jonas de Souza^2^, Adam L. Lock^2^, G. Andres Contreras^1*^

^1^Department of Large Animal Clinical Sciences, Michigan State University, East Lansing, Michigan, United States of America

^2^Department of Animal Science, Michigan State University, East Lansing, Michigan, United States of America

^*^Corresponding author

E-mail: [contre28@msu.edu](mailto:contre28@msu.edu)

| **Supplementary Table A.** Ingredient and nutrient composition of the close-up diet and the postpartum diet. | | |
| --- | --- | --- |
|  | Diet | |
|  | Close-up | Postpartum |
| Ingredient, % DM |  |  |
| Corn Silage | 42.0 | 29.7 |
| Alfalfa Silage | - | 10.9 |
| Alfalfa Hay | - | 12.4 |
| Grass Hay | 35.5 | - |
| Ground Corn | 7.09 | 19.6 |
| High Moisture Corn | - | 5.0 |
| Soybean Meal | 8.11 | 13.9 |
| Soyhulls | - | 1.90 |
| SoyChlor ^1^ | 2.52 | - |
| Amino acid supplement ^2^ | 1.13 | 1.42 |
| Fat supplement ^3^ | - | 1.21 |
| Mineral and vitamin mix ^4,5^ | 2.60 | 3.95 |
|  |  |  |
| Nutrient Composition, % DM ^6^ | |  |
| NDF | 38.5 | 29.8 |
| Forage NDF | 34.9 | 24.0 |
| CP | 14.6 | 16.7 |
| Starch | 17.2 | 24.6 |
| FA | 1.82 | 3.62 |
| 16:0 | 0.28 | 1.16 |
| 18:0 | 0.06 | 0.09 |
| *cis*-9 18:1 | 0.29 | 0.69 |
| *cis*-9, *cis*-12 18:2 | 0.82 | 1.27 |
| *cis*-9, *cis*-12, *cis*-15 18:3 | 0.17 | 0.15 |
| ^1^ West Central Soy, Ralston, IA.  ^2^ Spectrum Agriblue (Perdue Agribussiness, Salisbury, MD).  ^3^ Palmitic acid-enriched FA supplement (Nutracor; Wawasan Agrolipids, Johor, Malaysia). The supplement contained (g/100 g of fatty acid) 0.64 of C14:0, 84.5 of C16:0, 1.80 of C18:0, 7.88 of C18:1 *cis*-9, and 99.0% total fatty acids. Blend of Ca salts of palm FA supplement (Nutracal; Wawasan Agrolipids, Johor, Malaysia) and Palmitic acid-enriched FA supplement (Nutracor; Wawasan Agrolipids, Johor, Malaysia).  ^4^ Vitamin-mineral mix for the close-up diet contained (DM basis): 54.8% SoyChlor, 13.9% limestone, 10.0% rumen-protected choline, 8.8% di- calcium phosphate, 4.2% magnesium sulfate, 1.8% salt, 1.8% yeast, 4.4% trace minerals and vitamins, and 0.3% selenium yeast 600 (600 mg of Se/kg).  ^5^ Vitamin-mineral mix for the treatment diets contained (DM basis): 27.9% molasses, 15.3% limestone, 12.2% sodium bicarbonate, 11.8% blood meal, 8.7% dicalcium phosphate, 6.1% trace minerals and vitamins, 5.7% rumen-protected choline, 4.4% magnesium sulfate, 3.9% salt, 2.7% animal fat, 0.9% yeast, and 0.4% selenium yeast 600 (600 mg of Se/kg).  ^6^ Expressed as percent of as fed. | | |

| **Supplementary Table B.** Information of the genes of the lipolytic, lipogenic and glucose metabolism gene network. | | | |
| --- | --- | --- | --- |
|  | Gene | Full name | NCBI gene ID |
| Lipolysis network | | | |
|  | *ABDH5* | abhydrolase domain containing 5, comparative gene identification-58 (CGI58) | 535588 |
|  | *LIPE* | hormone sensitive lipase | 286879 |
|  | *LPL* | lipoprotein lipase | 280843 |
|  | *PNPLA2* | patatin like phospholipase domain containing 2, adipose triglyceride lipase (ATGL) | 508493 |
| Lipogenic network | | | |
|  | *ACACA* | acetyl-Coa carboxylase 1 alpha | 281590 |
|  | *FASN* | fatty acid synthase | 281152 |
|  | *AGPAT2* | 1-acylglycerol-3-phosphate O-acyltransferase 2 | 512112 |
|  | *DGAT1* | diacylglycerol O-acyltransferase 1 | 282609 |
|  | *DGAT2* | diacylglycerol O-acyltransferase 2 | 404129 |
|  | *ELOVL6* | ELOVL fatty acid elongase 6 | 533333 |
|  | *SCD1* | stearoyl-CoA desaturase 1 | 280924 |
| Glucose metabolism network | | | |
|  | *SLC2A4* | solute carrier family 2 member 4 or glucose transporter 4 (GLUT4) | 282359 |
|  | *PGK1* | phosphoglycerate kinase 1 | 507476 |

**Supplementary Table C.** Changes in body weight and body condition score in Holstein dairy cows at -11 ± 1 d prepartum (dry), and 11 ± 0.2 d (fresh) and 24 ± 0.4 d (lactation) after calving.

|  | Dry | Fresh | Lactation |
| --- | --- | --- | --- |
| Body weight (Kg) | 740 ± 13^a^ | 717 ± 13^b^ | 708 ± 12^b^ |
| Body condition score^1^ | 3.65 ± 0.07^a^ | 3.45 ± 0.07^b^ | 3.23 ± 0.06^c^ |

^abc^ Time-points with different letters differ significantly (*P* < 0.05).

^1^ Body condition score was assessed by three experienced technicians using a 5-point scale ^18^
